# Sleep macro- and microstructure in migraine and cluster headache: a systematic review of objective assessments

**DOI:** 10.1186/s10194-025-02252-4

**Published:** 2026-01-23

**Authors:** Veronica Munday, Ellie Watson, Diana Y. Wei, Alexander D. Nesbitt, Peter J. Goadsby, Philip R. Holland, Ivana Rosenzweig

**Affiliations:** 1 Sleep and Brain Plasticity Centre, Department of Neuroimaging, Institute of Psychiatry, Psychology and Neuroscience (IoPPN), King’s College London (KCL), London, UK; 2 Headache Group, Wolfson Sensory Pain and Regeneration Centre, Institute of Psychiatry, Psychology and Neuroscience (IoPPN), King’s College London (KCL), London, UK; 3 Department of Neurology, King’s College Hospital NHS Foundation Trust, London, UK; 4 Department of Neurology, Guy’s and St Thomas’ NHS Foundation Trust, London, UK; 5 Sleep Disorders Centre, Guy’s and St Thomas’ NHS Foundation Trust, London, UK

**Keywords:** Sleep, Polysomnography, Migraine, Cluster Headache

## Abstract

**Background:**

Patients with primary headache disorders including migraine and cluster headache often report poor sleep quality. While subjective reports of disturbed sleep are well established, the objective alterations in sleep architecture which occur in primary headache have not been systematically characterised. This systematic review aims to determine whether previously identified macrostructure changes in migraine occur across trigeminal autonomic cephalalgias, how sleep microstructure is altered, and how sleep architecture differs between the headache-free and headache attack phase.

**Methods:**

We conducted a systematic review across four databases (Embase, Medline, PubMed and APA PsycInfo) to identify studies assessing objective sleep parameters via polysomnography or actigraphy in individuals with migraine or cluster headache, including relevant animal models of migraine-related pain. Thirty studies met the inclusion criteria and were critically appraised in a narrative synthesis with respect to sleep macrostructure (including NREM-REM architecture, sleep fragmentation, sleep duration and sleep efficiency metrics), and sleep microstructure (cyclic alternating pattern and arousal indices).

**Findings:**

Significant heterogeneity across studies was observed resulting in varied findings. In adult migraine patients, 50% of included studies reported a significant reduction in sleep efficiency, while an increase in the number of awakenings was identified in the majority of studies reporting this metric, highlighting potential sleep fragmentation. Assessment of sleep microstructure identified altered arousal profiles, though no clear pattern of hypo- or hyper-arousal emerged. These arousal profiles were also found to differ across different phases of the headache attack, with a small number of studies identifying a reduction in arousal the night preceding migraine headache attack. Studies assessing sleep in cluster headache were less common, although some evidence indicates alterations to REM sleep architecture, consistent with previous findings in migraine. Sleep efficiency and sleep onset latency alterations further indicate sleep disruption however further replication is required to gain a broader understanding of how sleep is altered both in bout and in remission.

**Conclusion:**

Objective assessment supports disrupted sleep in headache disorders, with some evidence of altered sleep fragmentation and microstructure. Further studies focusing on these less widely reported metrics are necessary for a full understanding of sleep in migraine and cluster headache.

**Supplementary Information:**

The online version contains supplementary material available at 10.1186/s10194-025-02252-4.

## Introduction

A bidirectional relationship between sleep and headache is well noted [1], yet the specific nature of sleep alterations and the underlying mechanisms remain unclear. Sleep disturbance, including sleep deprivation and excessive sleep, is among the most frequently reported migraine triggers [2], and 80% of cluster patients report the onset of nocturnal sleep as a trigger when in bout [3]. Subjective sleep quality is also reported to be worse in migraine and cluster headache patients compared to headache-free controls [3–5], improving with time since attack [6], indicating that sleep disruption may be both a trigger and a symptom of primary headache.

Subjective assessment provides valuable insight into patient experience, but may be susceptible to confounding by mood, recall bias and pain anticipation. Polysomnography is the gold standard approach to evaluate sleep architecture, delineating non-rapid eye movement (NREM) sleep – comprised of N1, N2 and N3 substates which reflect increasing sleep depth – and rapid eye movement (REM) sleep [7]. Phasic events such as transient arousals and oscillations can be assessed to understand the fine microstructure of sleep.

A prior meta-analysis has begun to synthesise sleep macrostructure changes in migraine, highlighting reduced REM sleep in adult and paediatric patients, as well as broader sleep disruption in paediatric patients including reduced sleep onset latency and total sleep time, and elevated wake [4]. While this evidence supports sleep disruption in migraine, it does not determine whether sleep architecture changes are migraine-specific or characteristic of primary headache disorders more broadly, nor did it aim to assess how sleep architecture may differ in the headache-free and headache attack phase. Given significant reports of poor sleep quality in headache, the relative scarcity of significant macrostructure changes in adults may indicate more nuanced sleep alterations, such as altered fragmentation or arousals, may contribute to perceived poor sleep in headache patients. Thus, the aim of this systematic review is to synthesise the current evidence on objective sleep alterations in patients with migraine and trigeminal autonomic cephalalgias, and in relevant preclinical animal models to:

1. Advance understanding of sleep macrostructure changes in migraine and determine whether similar alterations occur in trigeminal autonomic cephalalgias.
2. Assess sleep microstructure alterations in migraine and trigeminal autonomic cephalalgias.
3. Determine whether sleep architecture differs across different phases of the headache attack.

## Methods

This review was conducted following the PRISMA 2020 guidelines. The protocol was registered on PROSPERO (CRD420250655569). The PROSPERO record initially included idiopathic intracranial hypertension (IIH); after screening yielded no eligible IIH studies and to improve focus on primary headache disorders, IIH was removed.

### Search strategy

Two reviewers (V.M. and E.W.) conducted an independent systematic search of the electronic databases PubMed, Embase (Ovid), Medline (Ovid) and APA PsycInfo (Ovid) from inception to November 7 2025. The following search terms were used: (migrain* OR cluster headache OR trigeminal autonomic cephalalgia OR TAC or trigeminovascular OR trigeminal OR headache*) AND (sleep* OR sleep disorder* OR NREM OR REM OR rapid eye movement OR slow wave) AND (electroencephalograph* OR polysomnograph* OR actigraph* OR EEG OR PSG). The search was limited to English-language studies.

### Eligibility criteria

Peer-reviewed clinical studies assessing objective sleep parameters in patients with primary headache disorders, diagnosed in accordance with the International Classification of Headache Disorders (ICHD), or validated preclinical animal models of migraine-related pain, were included (Table 1). Diagnosis in accordance with ICHD-1 [8], ICHD-2 [9] and ICHD-3 [10] were all included such that criteria were consistent with the relevant criteria at the time of publication or patient recruitment. Validated preclinical models of migraine-related pain include use of the clinical migraine trigger nitroglycerin [11], activation of the trigeminovascular system via application of inflammatory mediators to the dura, and use of headache-inducing agents such as umbellone [12] in combination with priming models [13, 14]. For the purpose of this review, we limited primary headache disorders to those with the strongest hypothalamic-brainstem sleep-pain interface in the current literature (migraine and trigeminoautonomic cephalalgias (TACs)). As the pathophysiological mechanisms underlying tension-type headache (TTH) remain relatively unknown, studies investigating sleep in TTH were excluded. Eligible studies assessed sleep architecture using validated objective methods – polysomnography (PSG), electroencephalography (EEG) or actigraphy. Cross-sectional and longitudinal observational designs, including both case-control and within-subject interictal-ictal comparisons were included. Studies focusing exclusively on sleep-disordered breathing, without inclusion of broader polysomnography measures such as total sleep time or sleep efficiency to allow assessment of how sleep apnoea measures influence overall sleep architecture, were excluded.

**Table 1:** The PICOS statement

Component of Question	Example
Population	Patients diagnosed with a primary headache disorder in accordance with the international classification of headache disorders.
Intervention	No intervention.
Control	Healthy controls or patients in the interictal headache phase.
Outcome	Objective sleep architecture in migraine, cluster headache or other primary headache disorders.
Study Design	Observational, descriptive, longitudinal, retrospective, prospective, cross-sectional, case-control and cohort studies.

### Study selection and data extraction

Titles and abstracts were independently screened by two reviewers (V.M. and E.W.). Eligible texts (Table 2), or those where eligibility was unclear were assessing for inclusion by one reviewer (V.M.). 25% of texts, determined via random number generator, were independently screened by a second reviewer (E.W.). The following data was extracted from each eligible full text: study design, year, patient demographics, headache diagnosis, medication use, objective sleep assessment, sleep macrostructure and microstructure findings, headache phase and limitations (Additional File 1).

**Table 2:** Inclusion and exclusion criteria

Features	Exclusion criteria	Inclusion criteria
**Manuscript characteristics**	• Review articles, including systematic reviews, case reports, conference proceedings, pre-prints, unpublished data, government reports, guidelines, statements, comments, dissertations and theses. • Studies employing an intervention where a comparison of baseline characteristics cannot be extracted. • Articles not in English.	• Original research articles • Longitudinal, cross-sectional, retrospective, prospective, observational, case-control or cohort studies investigating objective measures of sleep architecture in primary headache disorders.
**Patient diagnosis and recruitment**	• No use of diagnostic criteria. • Headache secondary to other condition. • Studies which include multiple headache disorders where data for an individual disorder cannot be extracted. • Diagnosis of Tension-Type Headache.	• Clinical diagnosis of a primary headache disorder according to the international classification of headache disorders (ICHD). • Where studies focus on patients with co-morbid sleep disorders this should be diagnosed in accordance with the international classification for sleep disorders (ICSD). • Animal studies using a validated preclinical migraine-relevant model
**Study Design**	• Either no case-control comparison or no ictal versus interictal comparison.	• Case-controlled studies • Observational cohort studies including the interictal and ictal headache phase. • Cross-sectional studies comparing patients in the interictal and ictal headache phase.
**Outcome Measures**	• Subjective assessment of sleep quality. • Studies exclusively assessing sleep apnoea measures.	• Objective assessment of sleep architecture for example using polysomnography or actigraphy.

### Quality assessment

Two reviewers (V.M. and E.W.) independently quality assessed all studies. Discrepancies were resolved through consensus, and where required, consultation with a senior reviewer. Human observational studies were assessed using the Effective Public Health Practice Project (EPHPP) tool [15]. Animal studies were assessed using the SYstematic Review Centre for Laboratory animal Experimentation (SYRCLE) risk of bias tool [16]. For EPHPP, studies were rated as strong, moderate or weak across 6 categories. As no intervention was employed, where the assessor was blinded to headache diagnosis during analysis of sleep architecture, studies were scored as strong for blinding to differentiate from studies where blinding was unclear. An overall rating was determined based on the following criteria: strong – no weak ratings and at least 2 strong ratings; weak – two or more weak ratings. All other studies were rated as moderate. No global score was produced for SYRCLE.

### Data synthesis

Due to the heterogeneity in study designs and outcomes, a narrative synthesis was conducted. Quantitative synthesis was considered but not performed due to outcome heterogeneity, non‑comparable metrics, and suspected sample overlap. Findings were first stratified based on sleep assessment (PSG or actigraphy). Within each assessment method, results were grouped according to primary headache disorder (migraine or cluster headache) and further separated into adult and paediatric populations. Sleep architecture metrics derived from PSG were classified into microstructural and microstructural indices. All indices are summarised in Table 3.

**Table 3:** Sleep architecture metrics. Definitions for key sleep architecture metrics reported across the included studies, categorised into those which are recorded in polysomnograpy or actigraphy, and whether they represent measures of sleep macrostructure or sleep microstructure

Sleep Architecture Metric	Subtype	Description
Polysomnography	Macrostructure and Microstructure	Gold standard approach for the assessment of sleep architecture using electroencephalography (EEG), electro-oculograph (EOG) and electromyography (EMG) alongside measurements of heart rate, blood oxygen levels and breathing
Actigraphy	Macrostructure	Assessment of sleep-wake architecture using accelerometer data, usually wrist-worn. Sleep-wake is estimated from activity counts, with periods of inactivity associated with sleep
Total Sleep Time (TST)	Polysomnography/Actigraphy – Macrostructure	Total time spent in all sleep states across the recording period
Time in Bed (TIB)	Polysomnography/Actigraphy – Macrostructure	Total duration from when participant gets into bed with the intention of sleeping, to when they get out of bed the following morning, often denoted from time of lights off to lights on
Sleep Efficiency (SE)	Polysomnography/Actigraphy – Macrostructure	Total sleep time divided by time in bed, expressed as a percentage
NREM	Polysomnography - Macrostructure	Non-rapid eye movement sleep. NREM sleep can be subdivided into 3 stages: N1, N2 and N3. Characterised in polysomnography by high power within the delta band (0.5-4Hz)
N1	Polysomnography - Macrostructure	NREM Stage 1 – Lightest stage of sleep representing the transition from wake into sleep
N2	Polysomnography - Macrostructure	NREM Stage 2 – Characterised by the occurrence of sleep spindles and K-complexes. The largest proportion of TST is spent in N2 in adults
N3	Polysomnography - Macrostructure	NREM Stage 3 – Deepest NREM sleep stage. Characterised by low frequency, high amplitude EEG. Also known as slow wave sleep. Older studies may report both N3 and N4, however modern scoring criteria consider only N3
REM	Polysomnography - Macrostructure	Rapid eye movement sleep. Desynchronised low amplitude, mixed frequency EEG with muscle atonia and intermittent occurrence of phasic eye movements
Wake after sleep onset (WASO)	Polysomnography/Actigraphy – Macrostructure	Total amount of wake recorded from initial sleep onset until the end of the recording
Sleep Onset Latency (SOL)	Polysomnography/Actigraphy – Macrostructure	Time from the beginning of the recording (lights off) to the beginning of the first sleep epoch
REM Latency	Polysomnography - Macrostructure	Time from the first epoch of sleep till the first epoch of REM
Awakenings	Polysomnography/Actigraphy - Macrostructure	Number of interruptions to sleep resulting in wake. Sometimes reported as awakenings per hour
Sleep stage transition index	Polysomnography – Macrostructure	Number of sleep stage transitions per hour. A higher value indicates more fragmented sleep
Arousal Index	Polysomnography - Microstructure	An arousal is an abrupt shift in EEG frequency include higher frequencies such as theta, alpha and gamma, which lasts at least 3 seconds, and follows at least 10-seconds of stable sleep. Arousal index is the number of arousals per hour of sleep
Periodic Limb Movement Index (PLMI)	Polysomnography - Microstructure	Number of periodic limb movements per hour of sleep. Periodic limb movements are defined a series of 4 or more limb movements - an increase in EMG activity of more than 8uV above baseline, lasting 0.5 to 5 seconds - within 5 to 90 seconds
Apnea-Hypopnea Index (AHI)	Polysomnography - Microstructure	Number of apnoea and hypopnea events per hour of sleep. Apnoea = Greater than 90% reduction in airflow for more than 10 seconds. Hypoapnea = more than 30% reduction in airflow for more than 10 seconds
Cyclic alternating pattern (CAP)	Polysomnography - Microstructure	Marker of sleep instability in NREM sleep. Consists of two phases: Phase A – transient EEG activation lasting 2–60 seconds, interspersed by Phase B, periods of basal EEG activity lasting 2–60 seconds
CAP Rate	Polysomnography - Microstructure	The percentage of NREM sleep occupied by CAP sequences
CAP A1	Polysomnography - Microstructure	Phasic EEG patterns consisting of predominantly synchronised patterns such as K-complexes and delta bursts, with less than 20% of the phase duration associated with desynchronised EEG. CAP A1 metrics may be presented as the number of A1 cycles, rate or percentage of NREM spent in A1
CAP A2	Polysomnography - Microstructure	Mixture of slow and fast rhythms in the phasic EEG activation, with 20-50% desynchrony. CAP A2 metrics may be presented as the number of A2 cycles, rate or percentage of NREM spent in A2
CAP A3	Polysomnography - Microstructure	Predominantly desynchronised patterns of cortical activity such as low amplitude fast-rhythms, K-alpha complexes and polyphasic bursts, occupying more than 50% of the duration of the CAP Phase, associated with strong autonomic activation and arousal. CAP A3 metrics may be presented as the number of A3 cycles, rate or percentage of NREM spent in A3
CAP A Duration	Polysomnography - Microstructure	Total or average duration of CAP A phases. CAP A duration may also be reported as the total or average duration for each individual A state (A1, A2 and A3)
CAP B Duration	Polysomnography - Microstructure	Total or average duration of CAP B phases between consecutive CAP A phases
CAP Index	Polysomnography - Microstructure	Number of CAP cycles per hour of sleep. CAP index can be calculated for each A subtype individually
CAP Cycles	Polysomnography - Microstructure	A single CAP cycle consists of a Phase A and phase B period. CAP cycles refers to the number of complete cycles across the night.
CAP Sequences	Polysomnography - Microstructure	At least two CAP cycles, preceded by Non-CAP NREM sleep. Reported as the number of CAP sequences across the night
Delta-burst index	Polysomnography - Microstructure	Number of delta bursts per hour of sleep. Delta bursts are a sequence of delta waves lasting two or more seconds, exceeding background amplitude by at least one third. This pattern of activity is similar to what may occur during CAP A1 but is calculated across both NREM and REM sleep
K-burst index	Polysomnography - Microstructure	Number of K-bursts per hour. K-burst consist of at least two consecutive K complexes. This pattern of activity is similar to what may occur during CAP A1 but is calculated across both NREM and REM sleep
Fast arousal index	Polysomnography - Microstructure	Abrupt shift in EEG frequency to include fast frequencies such as theta and alpha, lasting 3–30 seconds, following at least 10-seconds of sleep
Sleep spindles	Polysomnography - Microstructure	Transient burst-like oscillatory activity between 10-15Hz, generated by thalamocortical circuits which are the hallmark of N2 sleep
Sleep regularity index	Actigraphy – Macrostructure	Measure of the regulatory of sleeping and waking times across days of recording
Area under the curve - sleep	Actigraphy – Macrostructure	The amount of activity detected between sleep onset and sleep offset
Sleep over daytime	Actigraphy – Macrostructure	Fraction of epochs identified as sleep during the day
Sleep duration	Actigraphy – Macrostructure	Number of minutes asleep during time in bed
Motor activity index	Actigraphy – Macrostructure	Percentage of epochs during time in bed with an activity score of greater than 0

A summary heat map illustrating the relative consistency of findings across polysomnography studies is presented in Fig. 3 (Tables S1–S2). We synthesised direction of effects using a vote-counting approach consistent with synthesis without meta-analysis reporting guidelines, given heterogeneity in outcome metrics and suspected sample overlap in some cluster studies [17]. When ≥ 50% of studies reporting a metric demonstrated consistent findings in the same direction, a score of +2 (elevated) or − 2 (reduced) was assigned. When some studies reported a change, but findings were inconsistent across studies, or 3 or fewer studies reported the measure, a score of + 1 or − 1 was assigned to indicate partial evidence of alteration. A sub-analysis was conducted to consider heterogeneity in sample size, in which studies with a sample size less than 25 were excluded from vote-counting. The threshold of 25 was selected as this represents the lower quartile of sample sizes for the included case-control polysomnography studies.

## Results

### Overview of included studies

**Fig. 1:**
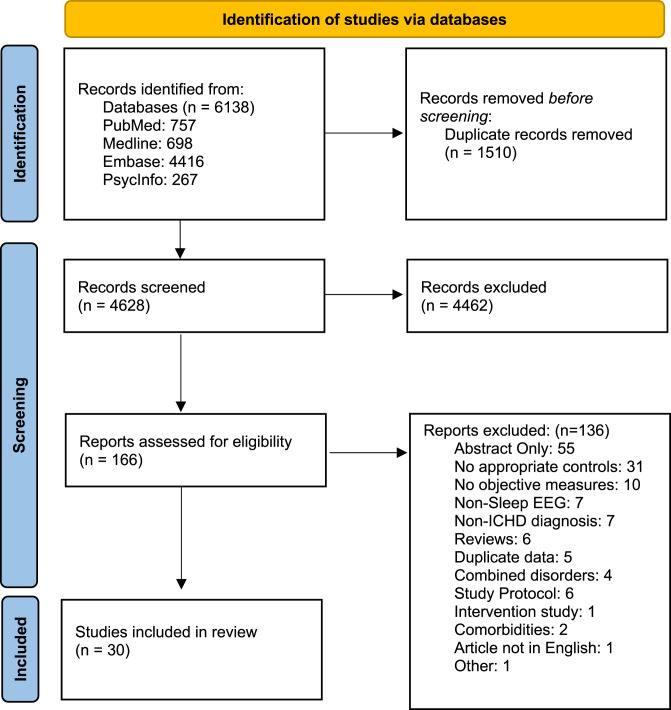
PRISMA flow diagram of the study selection process

**Fig. 2:**
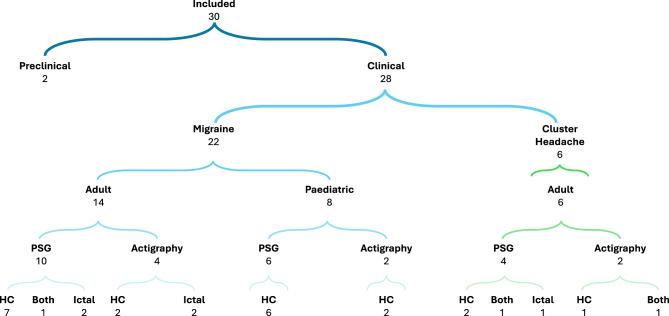
Summary of included studies detailing headache disorder, age of population, key methodology, and main comparator groups. Abbreviations: PSG, polysomnography; HC, Healthy Controls

Overall, 30 studies were included, comprising 28 clinical studies and 2 preclinical studies (Fig. 1). The majority focused on migraine; 14 primary research articles on adult populations and 8 investigating paediatric cohorts. A further 6 studies analysed adult cluster headache patients. PSG and actigraphy were conducted across both headache types, including case-control and headache phase specific analyses, as summarised in Fig. 2. No studies investigating other included primary headaches were identified in our search. Consequently, the discussion and conclusions of this review pertain predominantly to migraine and cluster headache.

Comprehensive tables of quality assessment scores are presented in the Additional File 2 and Supplementary Figures S1 and S2. The majority of studies were assessed using EPHPP, with 7 receiving strong global ratings, 3 weak and 18 rated moderate. Most studies recruited patients from exclusively from clinical settings. Studies which recruited participants across a wider range of settings were scored as strong for selection bias. Owing to their observational nature, most study designs were rated as moderate. Strong ratings were awarded where confounds beyond age, gender and body mass index (BMI) were controlled and weak ratings were awarded where these were not accounted for. No associations between quality assessment scores and the outcome of findings were identified. Nonetheless, several research groups contributed multiple publications included in this systematic review, potentially introducing bias related to shared methodologies, analytical approaches, or overlapping clinical databases.

### Sleep macrostructure – polysomnography findings

#### Adult migraine

Assessment of sleep architecture in adult migraine patients reveals considerable heterogeneity across studies. A detailed summary of study characteristics and findings can be found in Table S3. Among the eight studies reporting sleep macrostructure parameters, six had been previously included in a published meta-analysis. Of the two additional studies [18, 19], one assessed REM sleep and reported a significant reduction in migraine patients compared to controls, consistent with earlier findings [19]. Both studies identified decreased sleep efficiency in migraine patients, a finding which emerged as the most consistently reported across the included studies which report this measure (4/8, Table S1) [18–21]. In two studies reduced sleep efficiency was accompanied by an increase in time in bed [20, 21], which was also identified in a third study [22], while only one study reported decreased total sleep time [19]. Thus, changes in sleep efficiency may reflect an increase in sleep period, rather than a reduction in sleep duration. However, interpretation is limited by inconsistent reporting of time in bed and the use of restricted sleep windows in some studies [20, 23], limiting conclusions regarding habitual sleep pattern differences.

**Fig. 3:**
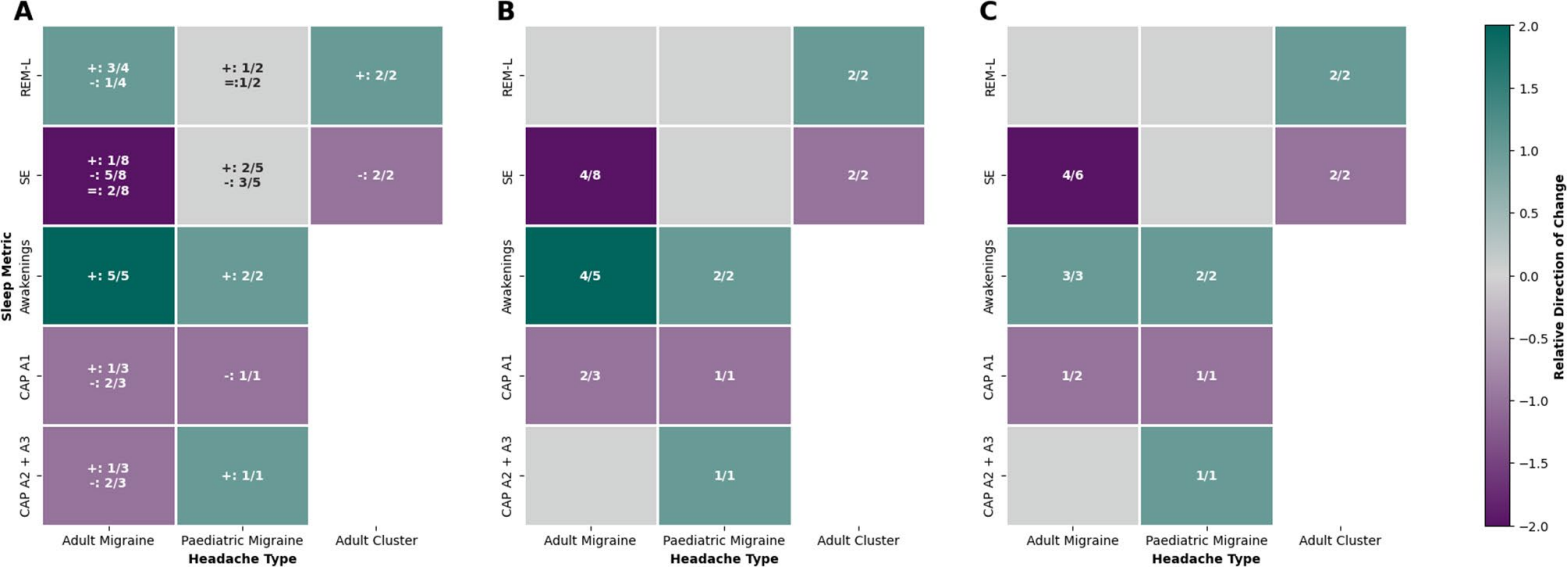
Summary of polysomnography changes in case-control studies. **A**) Heatmap showing directionality of changes in polysomnography-assessed sleep metrics in migraine and cluster headache compared to controls. Colours indicate the direction and consistency of change assessed via vote-counting. Dark purple = consistent decrease (≥50% studies), light purple = some evidence of decrease, light grey = no clear change, light teal = some evidence of increase, dark teal = consistent increase (≥50% studies). Numbers in each cell indicate studies reporting a difference / total studies assessing metric. + elevated in headache; - reduced in headache; = no change. **B**) Heatmap showing directionality of statistically significant changes in polysomnography-assessed sleep metrics in migraine and cluster headache. **C**) Sub-analysis including studies with ≥25 participants. Metrics: REM-L = REM latency; SE= sleep efficiency; CAP A1 and CAP A2+A3 = sleep microstructure (A1 stabilising; A2+A3 destabilising)

Sleep fragmentation may also contribute to poor sleep quality; however, measures are less widely reported. Only 3 studies reported wake after sleep onset [19, 22, 24], with one identifying a significant increase [19]. Similarly, 6 studies report awakenings, 4 of which identified a significant increase in migraine [19, 20, 22, 24], while the two which did not have small sample sizes, suggesting sleep instability should be considered more widely in future studies (Fig. 3).

Macrostructural changes in NREM and REM architecture reveal heterogeneity. Wu et al., identified a significant increase in N1 percentage, coupled with a decrease in N3 percentage in a study of 25 migraine without aura (MwoA) patients [19], suggesting a shift to increased light sleep and less restorative slow wave sleep. These findings were echoed in a previous study [21], however, two investigations by Engstrom and colleagues reported a significant increase in N3 sleep [22, 24], thereby underscoring the complexity of interpreting such alterations. Failure to stratify patients may contribute to heterogeneity. For instance, comparison of patients who predominantly experience migraine associated with sleep or awakening (sleep-related migraine, SM) to those with no clear sleep association (non-sleep-related migraine, NSM) showed conflicting findings. SM patients were found to have significantly elevated N1 versus controls (SM: 35 ± 17 mins, Co: 27 ± 19 mins) while NSM showed significantly elevated N3 compared to both controls (NSM: 104 ± 28 mins, Co: 86 ± 31 mins) and SM (SM: 88 ± 22 mins) [22]. While this exploratory study is limited in size it provides potential insight into variability which could be considered in future studies.

Notably, most included studies recruited patients primarily from tertiary centres, with a relatively high frequency of attack. A population study, with lower average attack frequency, failed to identify any significant sleep architecture changes compared to controls [25], indicating a need to ensure generalisability of findings across the breadth of the migraine population.

#### Paediatric migraine

Similar heterogeneity in findings was noted across paediatric migraine as detailed in Table S4. No additional studies have been conducted following on from a previous meta-analysis. Total sleep time was found to be reduced in three out of four studies which reported this measure [26–28]. Similarly to adult studies, only two studies report awakenings [27, 28], with both identifying a significant elevation, and wake after sleep onset was found to be significantly elevated in one study [26], suggesting sleep fragmentation may be an important factor which has been overlooked to date (Fig. 3). Additionally, one study identified a significant increase in sleep stage transition index in migraine versus controls, further indicating sleep fragmentation [26]. Periodic limb movements were found to be elevated in two studies, potentially contributing to fragmentation [26, 27], while increased bruxism was noted in one study [29].

#### Cluster headache

Polysomnography in cluster headache has been less widely explored (Table S5). In bout, sleep onset latency has been shown to be elevated, and sleep efficiency reduced compared to controls [30, 31], although potential overlap between participants in these two studies means further studies are warranted to replicate this. REM architecture may be altered, with elevated REM latency [31, 32] and decreased REM sleep noted (Fig. 3) [30]. Two further studies did not identify a significant difference in REM, but their smaller sample size may contribute to this heterogeneity. However, both identified an increase in N1 sleep during the cluster bout, especially on nights with headache [32, 33].

### Sleep macrostructure – findings from actigraphy

#### Adult migraine

Actigraphy studies provide an avenue for longitudinal assessment of sleep across different headache phases, accounting for habitual sleep patterns, but have been underutilised thus far (Table S6). No significant differences in sleep architecture were noted in a single study of chronic migraine patients, despite significant disruption when using subjective measures in the same cohort [34], highlighting discrepancies between subjective and objective approaches. Nevertheless, a second study assessing rest-activity cycles identified an increase in area under the curve during sleep, indicating elevated movement, and thus potential sleep fragmentation, as well as a reduction in inter-day stability, emphasising the potential for actigraphy to provide insight into habitual sleep changes [35]. Actigraphy also allows assessment of daytime sleep, of critical importance given the prevalent use of napping to mitigate ongoing migraine attacks. In chronic migraine, an elevated fraction of sleep over daytime supports increased napping compared to controls [35]. In a second study, longer nap duration was associated with decreased sleep efficiency the following night, potentially contributing to poor sleep quality and inter-day variability among chronic migraine patients [36].

#### Paediatric migraine

In paediatric patients, a similar scarcity of significant sleep architecture alterations is noted. Two studies identified a tendency towards elevated sleep onset latency in migraine [37, 38], contradicting some PSG findings [39]. A trend towards elevated wake after sleep onset was also seen, consistent with previous PSG, however this did not reach significance (Mi: 45.7 ± 19.1, Co: 35.7 ± 12.4, p = 0.059) [38]. As such, there is limited consensus across PSG and actigraphy findings in migraine.

#### Cluster headache

Two studies have utilised actigraphy in cluster headache populations, identifying increased time in bed in patients compared to controls, potentially indicating a perceived increase in sleep need [40, 41]. Consistent with PSG, sleep onset latency was found to be elevated in patients, both in bout and in remission [41].

### Sleep microstructure

#### Adult Migraine

Limited studies have explored sleep microstructure in migraine. Conflicting evidence supports both increased [18] and decreased [20, 22, 24] arousal index among migraine patients. One study identified a significant reduction in arousal index during REM sleep [21]. During NREM, cyclic alternating pattern (CAP) is defined by alternating phasic EEG events, delta bursts and K-complexes (A1) or moderate-to-high frequency arousals (A2, A3), interspersed with tonic background activity (B). Phase A1 is considered sleep preserving, whereas A2 and A3 are associated with cortical activation [42]. Alterations in CAP are evident in migraine compared to headache-free controls although no concordant phenotype is apparent (Fig. 3). Two studies identify a reduction in CAP rate in adult patients [21, 23], whilst a third counteracts this [18]. Varying phases have been found to be impacted, with some evidence supporting a reduction in A1 [23] concomitant with an increase in A2 and A3 [18], while diverging evidence indicates reduced A2 and A3 [21]. Similar heterogeneity is apparent for phase A and phase B duration. Engstrom et al., identified a significant reduction in delta-bursts (A1-like) only in SM patients [22], similar to Della Marca et al. [23], which restricted inclusion to this subtype, while NSM showed reduced fast arousals [22], indicating a need for patient stratification in microstructure analysis, to build on these findings in small samples. Discrepancies in the inclusion of migraine with aura (MwA) [18] may also compound variability.

#### Paediatric migraine

Assessment of sleep microstructure is similarly limited in paediatric migraine patients. A single study has assessed cyclic alternating pattern, identifying a significant decrease in CAP rate, driven by a decrease in A1 index, while the relative proportion of A2 and A3 phases were actually found to be elevated [28]. Additionally, one further study identified an increase in arousal index, indicating elevated fast arousals [26], although arousal index in REM has also been shown to be decreased [28].

A single study has explored alterations in sleep spindles in paediatric migraine patients, identifying a significant increase in spindle amplitude, alongside a decrease in the frequency of fast spindles [43], highlighting N2 microstructure as an area of interest for future studies.

#### Cluster headache

Limited assessment of sleep microstructure has been conducted in cluster headache. Arousal index has been noted to be reduced in a single study [30], and in a subset of participants with chronic cluster headache or on nights with headache attack in a second small sample study [32], although not in episodic cluster patients [31], suggesting arousal changes may occur with cluster headache attack occurrence chronicity, however further investigations are necessary.

### Sleep architecture changes across headache phases

#### Adult migraine

Few studies examine sleep architecture across headache phases, with many failing to state whether participants are headache free during inclusion. Nevertheless, a significant reduction in sleep onset latency noted in pre-ictal patients compared to those more than 48-hours from a migraine attack [24], along with a significant reduction in arousal number the night preceding/of migraine compared to headache-free nights [44] could indicate elevated sleep pressure prior to migraine onset [45].

Furthermore, spectral analysis in a limited patient sample indicated reduced EEG power during slow wave sleep, included a specific reduction in beta power (15–26 Hz), indicating lower cortical arousal [44]. Further non-linear EEG analysis identified a significantly higher degree of complexity loss during the first two sleep cycles on a migraine night, potentially indicating enhanced EEG synchronisation, supporting a reduction in cortical activation [46]. Thus, initial evidence indicates arousal levels may be reduced in the days to hours preceding migraine onset.

Limited studies have assessed the temporal relationship between poor sleep and headache attack onset. Short sleep duration (<6.5 hours) was not found to be associated with headache occurrence in the subsequent day (day 0). Diary assessed low sleep efficiency (<90%) was significantly associated with higher risk of migraine on day 1 (OR = 1.39, 95% CI = 1.07–1.81), with a similar pattern for actigraphy-assessed sleep efficiency, although the confidence interval overlapped 0 (OR = 1.17, 95% CI = 0.88–1.55). Therefore, the influence of poor sleep may develop over several days. Two days of short sleep duration (<6.5 hours) was not found to be a significant predictor of subsequent attacks, indicating that sleep fragmentation may be important rather than duration alone [36]. Assessment of sleep following migraine attack occurrence noted a small increase in sleep duration (7.3 minutes, 95% CI = 1.5–13.0) [47]. Thus, sleep architecture changes may be dependent on headache phase, rather than trait specific. Further studies are required to provide a broader understanding of phase specific sleep architecture alterations, potentially providing insight as to whether sleep alterations may be a trigger or early premonitory symptom of subsequent headache attacks.

#### Paediatric migraine

In paediatric patients, a significant reduction in motor activity index on nights preceding, of and following migraine compared to the headache-free interval indicates a similar decrease in arousability as noted above [37].

#### Cluster headache

Assessment of sleep architecture on nights with and without headache are comparatively limited in cluster headache. Neither polysomnography nor actigraphy studies have noted significant sleep architecture differences in bout compared to remission [31, 41]. Sleep onset latency has been shown to be elevated in remission compared to controls [41]. Reductions in REM sleep in bout may be driven more so by attack-free participants compared to individuals experiencing cluster attacks, as REM in headache free patients did not differ from controls [30]. Elevated wake and decreased sleep efficiency on attack-free nights compared to nights with cluster headache potentially indicate more widespread disruption on headache-free nights, but the small heterogeneous sample in this study should be noted [32]. Of note, arousal index was found to be lower on nights with cluster attack versus attack free nights [32], paralleling reduced arousal on nights preceding migraine. However, sample sizes are limited, requiring broader studies before robust conclusions can be drawn.

### Sleep architecture in animal models

As in patients, heterogeneity in findings was observed across the two included animal studies (Table S7). Yu et al., identified reduced sleep time and increased sleep onset latency in the 6-hours following nitroglycerin provocation [48], yet Lillo Vizin et al. identified no significant sleep architecture changes in the same model, nor following chronic nitroglycerin exposure [49]. Assessment of sleep architecture in two further migraine relevant models (dural inflammation and restraint stress priming followed by umbellone) identified no significant differences in immobility-defined sleep [49].

## Discussion

This systematic review aimed to synthesise the existing literature to determine whether a consistent pattern of sleep architecture changes occur across multiple primary headache disorders through assessment of both sleep macrostructure and microstructure in both migraine and cluster headache. While most studies show altered sleep in headache patients, findings remain inconsistent. Subtle changes in sleep architecture including fragmentation and alterations to arousals may contribute to poor sleep quality reported by headache patients. Alterations in sleep stability may occur at different phases of the headache attack, as opposed to being a stable phenotype. Although limited in number, studies indicate sleep alterations in cluster headache, with evidence of some similar changes to those previously reported in migraine.

### Sleep macrostructure in migraine

Migraine patients often report poor subjective sleep quality compared to headache-free controls, yet previous synthesis of sleep macrostructure identified a scarcity of significant sleep alterations among adult migraine patients. While less widely reported, several studies identified a significant increase in awakenings [19, 20, 24], suggesting sleep fragmentation may contribute to poor sleep quality, in both adult and paediatric migraine patients. Fragmentation may contribute to the reduction in sleep efficiency noted in half of the studies, occurring concomitantly in two [19, 20], but not reported in the others. Previous literature indicates that sleep fragmentation may contribute to subsequent migraine risk, as low sleep efficiency but not low sleep duration influenced subsequent migraine occurrence [36], while estimation of sleep time based on mobile phone use also suggests deviation from normal sleep and interrupted sleep increase subsequent headache risk [50]. This is consistent with findings across the pain field, which suggest sleep fragmentation, rather than sleep deprivation can lead to an increase in spontaneous pain and reduction in pain inhibition [51]. Fragmentation may implement wake-promoting neurotransmitter systems in the brainstem and hypothalamus, where activation and functional connectivity has been shown to be altered during migraine attacks [52–54], in the link between sleep and migraine. Elevated noradrenaline has been shown to precede transitions from NREM to wake, and microarousals [55–56], and altered locus coeruleus functional connectivity has been noted in co-morbid migraine and insomnia [57], highlighting the brainstem as a key region of pathophysiological interest.

Recent studies support previous REM sleep alterations, yet NREM findings remain heterogeneous. Elevated N1 and reduced N3 sleep reported in some studies align with poor sleep quality [19, 21], however, increased N3 has also been observed [24]. Patient stratification may compound this. Up to 42% of migraine patients report more than 75% of their attacks occurring during sleep or upon awakening [58]. A preliminary study showed sleep-related migraine patients exhibit non-restorative sleep, while non-sleep related demonstrated elevated N3 [22]. While sleep-related migraine is not an ICHD-defined criteria, further stratification in line with diagnosis of migraine with and without aura would benefit. Cortical spreading depression (CSD), the physiological correlate of migraine aura is increased following sleep deprivation in animal model, and the occurrence of CSD can result in elevated NREM and reduced REM [59–61]. Thus, there is a significant gap in determining whether sleep differs in MwA and MwoA, especially around the headache attack.

### Sleep microstructure alterations

Sleep microstructure is reported to be significantly altered in migraine. However, it is unclear whether this represents an overall reduction in arousals [21, 23] or a shift in proportion from CAP A1 to a greater CAP A2 and A3 [18, 28]. An overall decrease may indicate hypoarousability, however, this distinction is critical, as CAP A1 plays a role in sleep stabilisation, particularly during transitions into deeper NREM consolidated N3 sleep, while CAP A2 and A3 are linked to EEG desynchronisation and heightened arousal [42]. Thus, a reduced CAP rate driven by diminished A1 may reflect impaired arousal-buffering rather than enhanced NREM stability. This may explain the paradoxical finding of increased awakenings alongside reduced arousal index in some studies [20, 24, 28]. Notably, CAP A1 or delta-bursts were reduced specifically in sleep-related migraine [22, 23], where elevated N1 sleep and awakenings were also observed [22], consistent with a sleep-stabilising role of CAP A1. Broader CAP analyses are required to determine whether changes reflect hypoarousability or a predisposition to fragmentation. It remains uncertain whether these changes are migraine-specific or represent a broader phenotype, as CAP has not yet been assessed in other primary headache disorders.

### Sleep architecture in cluster headache

Assessment of sleep architecture in cluster headache is more limited, nevertheless, evidence supports sleep disruption. Notably, two studies indicate alterations in REM architecture [30, 31], mirroring previously reported changes in migraine [4], while sleep efficiency may also be reduced in both primary headache disorders. REM sleep dysregulation has been proposed previously in cluster headache, with initial hypotheses suggesting REM-linked attack occurrence, although the literature does not support this [32, 62]. Given this association, REM disruption may be expected to be worse on nights with headache, yet one study suggested REM sleep reduction was driven more so by patients without headache occurrence [30]. Additional evidence of reduced wake and improved sleep efficiency on nights with headache may indicate re-normalisation of sleep following attack occurrence. Cluster patients who report sleep as an attack trigger often report worse subjective sleep than those for who it is not a trigger [3]. Thus, further studies to assess whether attack anticipation contribute to poor sleep would be beneficial. Given the small number of studies, and especially given the small sample size, further replication is required before conclusions regarding sleep quality with versus without headache can be drawn.

Comparisons of sleep architecture in bout compared to remission suggest sleep disruption may continue into the remission period, as no differences were noted and sleep onset latency elevation persisted. Subjective sleep quality has previously been shown to improve with time since last attack, highlighting this as an area which requires future research [6].

Assessment of sleep microstructure in cluster headache is limited, although two studies indicate a reduction in arousal index, indicating that dysfunction of the ascending arousal system may contribute to sleep alterations across primary headache disorders.

### Across the headache cycle

Polysomnography studies often omit reporting of headache status. Where interictal-headache comparisons are conducted, headache-free controls are often absent, limiting interpretation. A small number of studies indicate decreased arousal or cortical activation on nights preceding or with migraine, compared to headache-free nights. Fatigue is a commonly reported premonitory symptom [63]; one hypothesis may be that reduced arousal represents an increase in associated sleep pressure in the lead up to an attack. Assessment of EEG power spectra, particularly the delta band would provide further insight into sleep homeostasis in headache patients [64]. Without comparison to controls, further studies are required to determine whether this reflects an overall decrease in arousal or a re-normalisation of arousal mechanisms prior to migraine onset. Outside of sleep, resting state EEG complexity has been shown to differ from controls in the interictal, but not the preictal phase [65], while impaired habituation to sensory stimuli also improves prior to attack [66, 67]. A role for thalamocortical circuitry has been implicated in this [68]. Given evidence of altered cortical arousals and initial evidence supporting altered sleep spindles in paediatric migraine [43], thalamocortical circuits may contribute to sleep architecture changes in migraine.

### Animal models

Animal models can be utilised to determine underlying pathophysiological mechanisms where they accurately recapitulate patient phenotypes. The findings of Yu et al. partially recapitulate some changes seen in paediatric migraine patients, namely decreased TST, and sleep onset latency changes noted in cluster headache [48], however there is significant heterogeneity in animal findings. The exclusive use of male mice by Yu et al is an important consideration given the female predominance of migraine [69]. No sleep architecture alterations were identified in a range of models using immobility defined sleep, however this precludes assessment of NREM-REM, a significant limitation given evidence of a potential reduction in REM sleep. Limited assessment of sleep fragmentation and power spectra hinders a comprehensive understanding of sleep alterations in animal models.

### Limitations

Interpretation is constrained by methodological variability. Inconsistent inclusion of some sleep parameters including awakenings, wake after sleep onset and arousal index precludes assessment of consistency across studies. Use of pre-defined sleep windows [20, 23, 43] and omission of adaptation nights [19–22, 24, 30] may yield polysomnography data which does not represent habitual sleep. Elevated time in bed in some migraine and cluster headache studies underscores this concern. Clear reporting of headache status is necessary, as altered arousal was noted across headache phases, while arousal index findings were among the most heterogeneous. Transparency in continued medication use is necessary, as some migraine prophylaxis including propranolol and amitriptyline have known effects on sleep, particularly REM [70–72].

Most studies recruited from tertiary headache centres, often with a relatively high attack frequency, limiting generalisability. The sole population-based study, where over half had a migraine frequency of less than once per month, reported no sleep architecture alterations [25]. Subjective reports indicate that sleep complaints increase with headache frequency, highlighting the need for further research in this domain [5]. Furthermore, included studies represent patients across a small number of clinical databases which should be expanded to further reproducibility.

Actigraphy remains underutilised in headache research. Concordance with polysomnography in cluster headache such as elevated SOL supports its utility [30, 31, 41]. Findings in migraine are less consistent, in part due to population differences (chronic versus episodic migraine). While polysomnography is essential for nuanced assessment of NREM, REM and arousals, actigraphy enables longitudinal evaluation across interictal and headache phases. Methodological harmonisation including patient stratification, medication and co-morbidity reporting, and headache attack status is needed to improve consistency.

## Conclusions

Objective assessment of sleep architecture provides evidence of the comorbidity between headache disorders and sleep disruption, with some evidence of similar sleep alterations across both migraine and cluster headache; however, a clear headache-associated sleep biomarker is not yet evident. Methodological differences contribute to a large degree of the heterogeneity seen in studies published to date. Evidence suggests sleep fragmentation and microstructural changes should be investigated more broadly, both during the interictal phase as well as in the lead up to and during headache occurrence, to gain a fuller understanding of sleep architecture changes in primary headache disorders.

## Supplementary Information

Below is the link to the electronic supplementary material.

Supplementary Material 1: Additional File 1: Summary of polysomnography metrics in case-control studies and data extraction tables

Supplementary Material 2: Additional File 2: Risk of bias assessment

## Data Availability

No datasets were generated or analysed during the current study.

## Abbreviations

BMI: Body mass index
CAP: Cyclic alternating pattern
CSD: Cortical spreading depression
EEG: Electroencephalography
EPHPP: Effective Public Health Practice Project
ICHD: International Classification of Headache Disorders
ICSD: International Classification of Sleep Disorders
MwA: Migraine with aura
MwoA: Migraine without aura
NREM: Non-rapid eye movement
NSM: Non-sleep-related migraine
OSA: Obstructive sleep apnoea
PSG: Polysomnography
REM: Rapid eye movement
SE: Sleep efficiency
SM: Sleep-related migraine
SOL: Sleep onset latency
SYRCLE: SYstematic Review Centre for Laboratory animal Experimentation
TAC: Trigeminal autonomic cephalalgia
TST: Total sleep time
TTH: Tension type headache
WASO: Wake after sleep onset
