# Supplementary material for: Sleep macro- and microstructure in migraine and cluster headache: a systematic review of objective assessments
Source: J Headache Pain. 2026 Jan 23;27(1):33. doi: 10.1186/s10194-025-02252-4 (PMC12857048; doi:10.1186/s10194-025-02252-4)
Supplement: Supplementary file 1 — Supplementary Material 1: Additional File 1: Summary of polysomnography metrics in case-control studies and data extraction tables [file 10194_2025_2252_MOESM1_ESM.docx]

| **Study** | **TST** | **TIB** | **SE** | **N1** | **N2** | **N3** | **REM** | **WASO** | **SOL** | **REM-L** | **Awk** | **SSTI** | **AI** | **NREM AI** | **REM AI** | **PLMI** | **AHI** |
| --- | --- | --- | --- | --- | --- | --- | --- | --- | --- | --- | --- | --- | --- | --- | --- | --- | --- |
| **Adult Migraine** | | | | | | | | | | | | | | | | | |
| Della Marca et al., 2006 [23] | - |  | - | - | - | - | - |  | - | - | - |  |  |  | ↓ |  |  |
| Engstrӧm et al., 2023 [22] | - | ↑NSM | - | ↑ SM | - | ↑NSM | - | - | - |  | ↑ SM |  | ↓NSM |  |  | - | - |
| Engstrӧm et al., 2023 [24] | - |  | - | - | - | ↑ | - | - | - |  | ↑ |  | ↓ |  |  |  | - |
| Karthik et al., 2013 [20] | - | ↑ | ↓ | - | - | - | - |  | ↑ | - | ↑ |  | ↓ | ↓ |  | - | - |
| Kristiansen et al., 2011 [25] | - |  | - | - | - | - | - |  | - | - |  |  | - |  |  |  |  |
| Nayak et al., 2016 [21] | - | ↑ | ↓ | ↑ | - | ↓ | - |  |  | - |  |  | - | - | ↓ | - | - |
| Wu et al., 2020 [19] | ↓ |  | ↓ | ↑ | ↑ | ↓ | ↓ | ↑ | ↑ | ↑ | ↑ |  |  |  |  | ↑ | ↑ |
| Zhou et al., 2023 [18] |  |  | ↓ |  |  |  |  |  |  |  |  |  | ↑ |  |  |  |  |
| **Paediatric Migraine** | | | | | | | | | | | | | | | | | |
| Armoni Domany et al., 2019 [39] | - |  | ↑ | - | ↑ | ↓ | - |  | ↓ |  |  |  | - |  |  |  |  |
| El-Heneedy et al., 2018 [26] | ↓ |  | ↓ | - | - | ↑ | ↓ | ↑ | ↑ | - |  | ↑ | ↑ |  |  | ↑ | - |
| Esposito et al., 2013 [27] | ↓ | ↓ | - | - | - | - | - | - | - |  | ↑ | - |  |  |  | ↑ | - |
| Masuko et al., 2014 [29] |  |  | - | - | - | - | - |  | - |  |  |  | - |  |  |  | - |
| Roccella et al., 2019 [28] | ↓ | ↓ | - | - | - | - | - | - | - |  | ↑ | - |  |  | ↓ | - | - |
| **Cluster Headache** | | | | | | | | | | | | | | | | | |
| Barloese et al., 2015 [30] | - |  | ↓ | - | - | - | ↓ |  | ↑ | ↑ |  |  |  |  |  | - | - |
| Lund et al., 2019 [31] | - |  | ↓ Bout | - | - | - | - |  | ↑ | ↑ Bout |  |  | - |  |  | - | - |
| Nobre et al., 2003 [33] |  |  |  | ↑ |  |  | - |  |  |  |  |  |  |  |  | - | - |

**Key: - Reported Metric; ↑ elevated in headache patients; ↓ decreased in headache patients**

**Table S1:** Summary of reported macrostructure polysomnography metrics in case-control studies. Summary of included metrics across each case-control polysomnography study reporting sleep macrostructure and arousal index. - represents metrics which were reported but not significantly different. ↓ represents metrics which were significantly lower in headache. ↑represents metrics which were significantly elevated in headache. NSM, SM and Bout provides further detail as to which subgroup showed significant differences compared to headache free controls. Abbreviations: AI, arousal index; AHI, apnoea-hypopnea index; Awk, awakenings; Bout, cluster headache patients in bout; NREM. Non rapid eye movement; NREM AI, non rapid eye movement; arousal index; NSM, non-sleep related migraine; PLMI, periodic limb movement index; REM, rapid eye movement; REM AI, REM arousal index, REM-L, REM latency; SE, sleep efficiency; SM, sleep-related migraine; SOL, sleep onset latency; SSTI, sleep stage transition index; TIB, time in bed; TST, total sleep time; WASO, wake after sleep onset.

| **Study** | **CAP Rate** | **A1 Index** | **A2 Index** | **A3 Index** | **A1 (n)** | **A2 (n)** | **A3 (n)** | **A1%** | **A2%** | **A3%** | **Mean A Dur** | **Mean B Dur** | **CAP Cycles** | **CAP Seq** | **D-burst Index** | **K-burst Index** |
| --- | --- | --- | --- | --- | --- | --- | --- | --- | --- | --- | --- | --- | --- | --- | --- | --- |
| **Adult Migraine** | | | | | | | | | | | | | | | | |
| Della Marca et al., 2006 [23] | ↓ |  |  |  | ↓ | - | - |  |  |  | - | ↑ |  | - |  |  |
| Nayak et al., 2016 [21] | ↓ | - | ↓ | ↓ |  |  |  |  |  |  | ↑ | ↓ | ↓ | ↓ |  |  |
| Zhou et al., 2023 [18] | ↑ | ↓ | ↑ | ↑ | ↓ | ↑ | ↑ | ↓ | ↑ | ↑ | ↑ | - | ↑ | ↑ |  |  |
| Engstrӧm et al., 2023 [22] |  |  |  |  |  |  |  |  |  |  |  |  |  |  | ↓SM | ↑NSM |
| Engstrӧm et al., 2023 [24] |  |  |  |  |  |  |  |  |  |  |  |  |  |  | - | - |
| **Paediatric Migraine** | | | | | | | | | | | | | | | | |
| Roccella et al., 2019 [28] | ↓ | ↓ | - | - |  |  |  | ↓ | ↑ | ↑ |  | - |  | ↓ |  |  |

**Key: - Reported Metric; ↑ elevated in migraine patients; ↓ decreased in migraine patients**

**Table S2:** Summary of reported microstructure polysomnography metrics. Summary of included metrics across each polysomnography study reporting sleep microstructure. – represents metrics which were reported but not significantly different. ↓ represents metrics which were significantly lower in migraine. ↑represents metrics which were significantly elevated in migraine. Abbreviations: CAP, cyclic alternating pattern; A1, CAP A1; A2, CAP A2; A3, CAP A3; CAP Seq, CAP sequences. D-burst Index, delta burst index; SM, sleep-related migraine; NSM, non-sleep-related migraine.

| **Study / Country (Ref)** | **Headache Diagnosis** | **N (Patients**  **[% Female] / Controls [%Female])** | **Sleep Assessment** | **Key Macrostructure Findings** | **Key Microstructure Findings** | **Medication Use** | **Limitations/ Bias Notes** |
| --- | --- | --- | --- | --- | --- | --- | --- |
| Della Marca et al, 2006  Rome, Italy  [23] | Episodic MwoA  (>5 attacks /month) | 10 [70%] / 10 [70%] | Video PSG (Lab)  ✓ CAP | No significant macrostructure difference | ↓ Cap Rate, ↓ CAP A1 number, ↓ REM AI  ↑ CAP Phase B duration | No prophylactic medication. Acute medication allowed, but all patients interictal. | Small sample size; restricted sleep window; sleep-related migraine patients |
| Engstrӧm et al., 2013  Trondheim, Norway  [22] | MwA and MwoA  (2-6 attacks /month) | 33 [75.8%] / 34 [58.8%]  SM: 15 [66.7%]  NSM: 18 [83.3%] | Ambulatory PSG (Lab)  ✓ Microstructure | **SM vs Controls:**  ↑ N1, Awakening index  **NSM vs Controls:**  ↑ TIB, ↑N3  **SM vs NSM:**  ↓ N3 | SM vs Controls:  ↓ Delta-burst index  NSM vs Controls:  ↓ Fast arousal index  ↑ K-burst index  SM vs NSM:  ↑ K-burst index | No prophylactic medication for 4 weeks. Acute painkillers and triptans in 5 patients in preceding 48-hours. | No adaptation night; medication use; subgrouping outside of ICHD criteria. |
| Engstrӧm et al., 2013  Trondheim, Norway  [24] | MwA and MwoA  (2-6 attacks / month) | Interictal 33 [75.8%] / Preictal 9 [66.7%] / Postictal 8 [87.5%] / Control 34 [58.8%] | Ambulatory PSG (Lab) | **Interictal vs Control:**  ↑ Awakening index, ↑ N3 sleep  **Preictal vs Interictal:** ↓ SOL preictally | Interictal vs Control:  ↓ Fast arousal index | No migraine prophylaxis for at least 4-weeks. Painkillers and triptans allowed. | No adaptation night; No ictal patients; No comparison between pre- post-ictal and controls. |
| Goder et al., 2001  Kiel, Germany  [44] | MwoA | 8 [75%] / 0 | PSG (Lab, up to 4 nights)  ✓ Spectral analysis | No significant macrostructure changes on migraine compared to control night. | ↓ arousals on nights with migraine attack.  ↓ Total power during SWS, ↓ beta 2 power (15-26Hz) during SWS.  No changes to REM power. | Use of acute medication for attack allowed during study (e.g. triptans). | Sleep-related migraine patients; Includes adaptation night; Small sample; No controls. |
| Karthik et al., 2013  Bangalore, India  [20] | MwoA | 30 [80%] / 32 [28.1%] | PSG (Lab) | ↓ SE, ↓ N4, ↓NREM,  ↑ TIB, ↑ Wake,  ↑ SOL,  ↑ Awakenings | ↓ Arousal index NREM | No prophylactic medication for at least 1 month. | Gender imbalance; No adaptation night; Restricted lights-on time |
| Kristiansen et al., 2011  Oslo, Norway  [25] | MwA and MwoA | MwA 38 [68.4%] / MwoA 71 [66.2%] / Controls 431 [39.2%] | PSG (Hospital) | No significant macrostructure differences. | None reported | Not reported | High prevalence of OSA; Descriptive statistics only; Incomplete demographics for subgroups; Low migraine attack frequency |
| Nayak et al., 2016  Bangalore, India  [21] | MwoA | 25 [76%] / 25 [76%] | PSG (ILab)  ✓ CAP | ↑ TIB, ↑ Wake, ↑N1,  ↓ SE, ↓N3 | ↓ Arousal Index REM, ↓ CAP rate, ↓ CAP cycles, ↓ CAP sequences, ↓ CAP Phase B duration, ↓ CAP A2 and A3 index  ↑ CAP Phase A duration, ↑ CAP A1 duration, ↑ Average CAP A2 and A3 phase duration | No long-term prophylaxis or medications known to interfere with sleep. | Inconsistency between table and text; no adaptation night |
| Strenge et al., 2001  Kiel, Germany  [46] | MwoA | 5 [80%] / 0  (Interictal vs Ictal comparison) | PSG (Lab, Non-linear EEG dynamics) | Not reported | ↓ extreme values for estimated correlation dimension in second SWS cycle. | Not reported | Overlap in sample with Goder et al.; Small N; Analysis restricted to 1^st^ 2 sleep cycles. |
| Wu et al., 2020  Zhengzhou, China  [19] | Migraine and Vestibular Migraine | 52 [55.8%] / 54 [55.6%] | PSG (Lab) | ↑WASO, ↑ SOL, ↑ REM Latency, ↑N1, ↑N2,  ↓TST, ↓SE, ↓ N3, ↓REM ↑ Awakening Index, | ↑PLMI, ↑ AHI | No drugs effecting sleep in week before PSG. | Unclear migraine MwA or MwoA; Unclear phase timing; No adaptation night; Large N; Inconsistent naming of awakening /micro-awakening index. |
| Zhou et al., 2023  Zhengzhou, China  [18] | MwA or MwoA and Vestibular Migraine | 33 [66.7%] / 30 [80%] | PSG (Lab)  ✓ CAP | ↓ SE | ↑ Arousal Index, ↓ CAP A1 index, ↑ CAP time, ↑ CAP cycles, ↑ CAP index, ↑ CAP Rate, ↑ CAP sequences, ↑ CAP A duration, ↑ CAP A2 index, ↑CAP A3 index | No ongoing migraine treatment. PSG in Interictal phase. | Limited macrostructure reporting; |

**Table S3:** Summary of adult migraine studies assessing sleep macrostructure and microstructure using polysomnography (PSG), including key cyclic alternating pattern (CAP) and spindle findings. ↓ = decreased in headache patients; ↑ = increased in headache patients versus controls. Abbreviations: AI, arousal index; CAP, cyclic alternating pattern; MwA, migraine with aura; MwoA, migraine without aura; NREM, non-rapid eye movement; NSM, non-sleep related migraine; PSG, polysomnography; REM, rapid eye movement; SE, sleep efficiency; SM, sleep-related migraine; SOL, sleep onset latency; SWS, slow wave sleep; TIB, time in bed; TST, total sleep time; WASO, wake after sleep onset.

| **Study/ Country (Ref)** | **Headache Type** | **N (Patients [%F]) / Controls [%F])** | **Sleep Assessment** | **Key Macrostructure Findings** | **Key Microstructure Findings** | **Medication** | **Limitations / Bias Notes** |
| --- | --- | --- | --- | --- | --- | --- | --- |
| Armoni Domany et al., 2019  Cincinnati, USA  [39] | MwA and MwoA | 185 [38.9%] / 180 [44.9%] | PSG (Lab) | ↓ SOL, ↓ N3,  ↑SE, ↑ N2 | None reported | 14% treated with amitriptyline, 20% with topiramate | Children referred to sleep clinic; Controls not matched for similar sleep disturbances; medication use; poor matching |
| El-Heneedy et al., 2019  Tanta, Egypt  [26] | MwA and MwoA | 40 / 20 | PSG (Lab) | ↓ TST, ↓ SE, ↓ REM  ↑ SOL, ↑ WASO, ↑ N3, ↑ Sleep stage transition index, ↑ REM without atonia index | ↑ Arousal Index, ↑ PLMI, ↑ Snoring index | Participants using medications which effect sleep excluded. Migraine medication not reported. | Incomplete demographics; No differentiation between migraine subgroups in analysis. |
| Esposito et al., 2013  Naples, Italy  [27] | MwoA | 34 [41.2%] / 51 [45.1%] | PSG (Lab) | ↓ TIB, ↓ TST,  ↑ Awakenings/h | ↑ PLMI | Migraine medication not reported. Anticonvulsant and Psychoactive medications excluded. | Includes adaptation night; Unclear phase timing; Restricted socioeconomic background |
| Masuko et al., 2014  Sao Paulo, Brazil  [29] | MwA and MwoA  (<4 attacks/ month) | 20 [50%] / 20 [50%] | PSG (Lab) | No significant macrostructure differences. | ↑ Bruxism | No regular medication use. | Small N; Well-matched for SBD. |
| Roccella et al., 2019  Palmero, Italy  [28] | MwoA | 33 [39.3%] / 52  [ 44.2%] | PSG (Lab)  ✓ CAP | ↓ TIB, ↓ TST,  ↑ Awakening/h | ↓ Arousal Index REM, ↓ CAP Rate, ↓ CAP sequences, ↑ CAP A1 mean duration, ↑ CAP A2 mean duration, ↓ CAP A1 index, ↓ Proportion CAP A1%, ↑ Proportion CAP A2%, ↑ Proportion CAP A3% | No prophylactic medication or any regular medication for proceeding 2 weeks. Interictal during PSG. | Includes adaptation night; Restricted socioeconomic background |
| Ulgen Temel et al., 2024  Gaziantep, Turkey  [43] | MwoA | 17 [70.6%], 15 [66.7%] | Video-PSG (Lab)  ✓ Sleep Spindles | None reported. | ↑ Mean, fast and slow spindle amplitude,  ↓ Fast spindle frequency | No migraine prophylaxis. Interictal phase during PSG. | Restricted sleep routine in days preceding PSG; Only first N2 episode analysed |

**Table S4:** Summary of paediatric PSG studies in migraine, highlighting differences in sleep architecture and emerging microstructural patterns. ↓ = decreased in headache patients; ↑ = increased in headache patients versus controls. Abbreviations: AI, arousal index; CAP, cyclic alternating pattern; MwA, migraine with aura; MwoA, migraine without aura; NREM, non-rapid eye movement; PLMI, periodic limb movement index; PSG, polysomnography; REM, rapid eye movement; SE, sleep efficiency; SOL, sleep onset latency; SWS, slow wave sleep; TIB, time in bed; TST, total sleep time; WASO, wake after sleep onset.

| **Study / Country (Ref)** | **Headache Type** | **N (Patients [% Female] / Controls [% Female])** | **Sleep Assessment** | **Key Macrostructure Findings** | **Key Microstructure Findings** | **Medications** | **Limitations / Bias Notes** |
| --- | --- | --- | --- | --- | --- | --- | --- |
| Barloese et al., 2015  Glostrup, Denmark  [30] | Episodic and Chronic Cluster Headache In bout. | 37 [24.3%] / 25 [36%] | PSG (Lab) | **CH vs Controls:**  ↓ SE, ↑SOL, ↑ REM Latency, ↓ REM | ↓ Arousals/h (especially in non-rhythmic CH patients) | Prophylactic medications kept stable for at least 7 days. Acute treatment with oxygen first, but other medication (triptans) allowed if required. Use of sleep medication paused during study. | No adaptation night; subgrouping based on rhythmicity and presence of attack during PSG. |
| Lund et al., 2019  Glostrup, Denmark  [31] | Episodic Cluster Headache (In-bout vs remission) | 32 [18.8%] + 23 [26.1%] / 25 [32%] | PSG (Lab) | **In-bout vs Cont.**  **↓** SE, ↑ SOL, ↑ REM Latency  **Remission vs Cont.**  ↑ SOL  **Bout vs Remission.**  No significant macrostructure differences. | No significant difference in arousals per hour. | Prophylactic medication kept stable for one week. Attacks preferably treated with oxygen. | Some possible participants overlap with Barloese et al; Included both paired and unpaired analysis bout vs remission. |
| Nobre et al., 2003  Rio de Janeiro, Brazil  [33] | Episodic Cluster Headache | 16 [12.5%] / 29 [10.3%] | PSG (Lab) | ↑ N1 sleep | None reported | Prophylactic medication discontinued 1-week prior. | Scoring criteria not reported; incomplete macrostructure reports; no descriptive statistics. |
| Zaremba et al., 2012  Essen, Germany  [32] | Episodic and Chronic Cluster Headache | 5 [0%) / 0  (Attack vs no attack) | PSG (Lab) | ↓ Wake, ↑ N1, ↑ SE on nights with headache.  **Chronic CH:**  ↓ Wake, ↓ N3, ↑ SE on nights with headache.  **Episodic CH:**  ↓ Wake, ↓ N2, ↑ N1, ↑ SE on nights with headache  **cCH vs eCH:**  ↓ Wake  ↓ TST and SE in eCH on nights without headache.  ↓ SOL in cCH patients on nights with headache vs eCH.  **Across all nights**  ↓ wake, ↓ N4, ↑ SE in cCH vs eCH | eCH: ↓ Arousal index on nights with headache  **cCH vs eCH:**  ↓ Arousal index in cCH | No prophylactic medication during the study. | Small N; Large number of comparisons; All patients in bout rather than bout vs remission. |

**Table S5:** Summary of polysomnographic data in patients with cluster headache, detailing macrostructural sleep disruption and phase-specific alterations. ↓ = decreased in headache patients; ↑ = increased in headache patients versus controls. Abbreviations: cCH, chronic cluster headache; CH, cluster headache; eCH, episodic cluster headache; PSG, polysomnography; REM, rapid eye movement; SE, sleep efficiency; SOL, sleep onset latency; TST, total sleep time.

| **Study / Country (Ref)** | **Headache Type** | **N (Patients [%Female] / Controls [%Female])** | **Sleep Assessment** | **Key Sleep Findings** | **Medication** | **Limitations / Bias Notes** |
| --- | --- | --- | --- | --- | --- | --- |
| David et al., 2022  Recife, Brazil  [35] | Chronic Migraine | 23 [95.7%] / 23 [95.7%] | 15-days actigraphy | ↑ AUC during sleep indicating fragmentation, ↑ Variability in sleep and wake times, ↑ Sleep over daytime, ↓ interday stability, ↓ sleep regularity index, ↓ circadian function index | Continued use of prophylactic (8.7%) and acute migraine treatments (87%) | Different actimeter brand; ethnicity differences between groups; few macrostructural sleep parameters reported; no assessment of days with vs without headache. |
| Ong et al., 2023  Chicago, USA  [34] | Chronic Migraine | 20 [100%] / 20 [100%] | 4-week actigraphy. | ↑ Days with naps. ↓ Diary SE but no significant difference in any actigraphy-measured sleep parameters.  ↑ Nap duration correlated with ↓ Sleep efficiency. Nap duration ↑ with headache severity. | Medication known to effect sleep exclusion criteria. | Continued medication use; Female only cohort; No differentiation between days with and without migraine headache; Diary / actigraphy discordance. |
| Bertisch et al., 2020  Boston, USA  [36] | Episodic MwA or MwoA | 98 [87.8%] / 0  Interictal-ictal | 6-week actigraphy | ↓ risk of headache following actigraphy-assessed high WASO and low SE on Day 0.  ↑ Risk of headache on Day 1 following diary assessed low SE. Risk elevated with actigraphy SE but CI overlapped 0.  No increase in headache risk following 2 days of short sleep duration. | 26% using daily migraine prophylactic medication | No control group to assess baseline disruption; Low levels of baseline sleep disruption in sample based on questionnaire. |
| Vgontzas et al., 2022  Boston, USA  [47] | Episodic MwA and MwoA | 98 [87.8%] / 0  Interictal-ictal | 6-week actigraphy | ↑ Sleep duration on nights following headache vs headache-free days.  SE and WASO did not differ. | 2/3 of attacks treated with acute medication | Same population as Bertisch et al.; No sub-analysis of with vs without aura; low to moderate sleep disruption and depression in population; no preictal vs post-ictal analysis. |
| Bruni et al., 2004  Rome, Italy  [37] | Paediatric MwoA  (>1 attack/week) | 18 [44.4%] / 17 | 2-week actigraphy | No significant sleep measures migraine vs control.  ↓ Motor activity index night preceding migraine attack, night during the attack and night following the attack compared to nights without an attack. | Not reported | Different recording length patient vs control; unclear gender matching |
| Nahman-Averbuch et al., 2022  Cincinnati, USA  [38] | Paediatric Migraine | 20 [85%] / 20 [85%] | 7 days actigraphy | No significant differences in actigraphy measures.  Trend towards ↑ WASO (p=0.059) and ↑ SOL (p=0.063). | Continued NSAIDs use. | Short recording period/ Unclear if headache-free throughout. |
| Lund et al., 2019  Glostrup, Denmark  [40] | Episodic Cluster Headache in bout and remission | 23 [21.7%] / 15 [26.7%] | 2-weeks actigraphy | ↑ TIB, ↑ TST in patients in bout vs controls.  No significant differences bout vs remission or remission vs controls. | Prophylactic medication stable through study. | Large data loss; demographics not separated bout vs remission; low frequency of attacks during recording period compared to reported at inclusion. |
| Ran et al., 2023 Stockholm, Sweden  [41] | Chronic and Episodic Cluster Headache in bout and remission | 32 eCH [40.6%] / 18 cCH [61.1%] / 42 [45.2%] | 2-weeks actigraphy | ↑ TIB, ↑ SOL in CH vs controls overall.  ↑ TIB, ↑ SOL, ↓ SE in cCH vs controls.  ↑ SOL in eCH vs controls  No significant difference eCH vs cCH  No difference patients in bout vs remission.  ↑ SOL, ↓ SE in bout vs controls.  ↑ SOL in remission vs controls | 18% eCH and 44.4% cCH taking medication which may affect sleep. | High co-morbidity in CH population; High inclusion of cCH patients |

**Table S6:** Summary of actigraphy studies across migraine and cluster headache. ↓ = decreased in headache patients; ↑ = increased in headache patients versus controls. Abbreviations: AUC, area under the curve; cCH, chronic cluster headache; CH, cluster headache; CI, confidence intervals; eCH, episodic cluster headache; MwA, migraine with aura; MwoA, migraine without aura; SE, sleep efficiency; SOL, sleep onset latency; TIB, time in bed; TST, total sleep time; WASO, wake after sleep onset.

| **Study / Country (Ref)** | **Animal Model** | **N (Model / Control)** | **Sleep Assessment** | **Key Macrostructure Findings** | **Limitations / Bias Notes** |
| --- | --- | --- | --- | --- | --- |
| Yu et al., 2023  Beijing, China  [48] | Acute Nitroglycerin | 7 [0%] / 7 [0%] | EEG | ↓ TST in the 6-hours following nitroglycerin injection compared to saline.  ↑ sleep latency following nitroglycerin | Only male mice; only 6-hours EEG recording; Limited reporting of sleep macrostructure measures; potential order effect – saline always first recording; short habituation to EEG recording chamber. |
| Lillo-Vizin et al., 2024  Arizona, USA  [49] | Acute nitroglycerin, chronic nitroglycerin, dural CGRP application, restraint stress priming + umbellone. | 4-7 / 6-7 per group. | EEG (acute and chronic nitroglycerin)  Immobility-defined sleep. | No significant difference in sleep macrostructure following acute or chronic NTG compared to vehicle-treated mice.  No significant difference in delta power between vehicle and nitroglycerin-treated mice.  No significant difference in immobility-defined sleep following dural application of CGRP.  No significant difference in immobility-defined sleep following restraint stress priming plus umbellone. | Immobility-defined sleep precludes analysis of NREM/REM distribution and EEG spectral power; low sample size in some groups. |

**Table S7:** Summary of animal model studies. ↓ = decreased in headache-relevant models versus controls; ↑ = increased in headache-relevant models versus controls. Abbreviations: CGRP, calcitonin gene-related peptide; EEG, electroencephalography; NREM, non-rapid eye movement; REM, rapid eye movement; TST, total sleep time.
