# Supplementary material for: Sleep macro- and microstructure in migraine and cluster headache: a systematic review of objective assessments
Source: J Headache Pain. 2026 Jan 23;27(1):33. doi: 10.1186/s10194-025-02252-4 (PMC12857048; doi:10.1186/s10194-025-02252-4)
Supplement: Supplementary file 2 — Supplementary Material 2: Additional File 2: Risk of bias assessment [file 10194_2025_2252_MOESM2_ESM.docx]

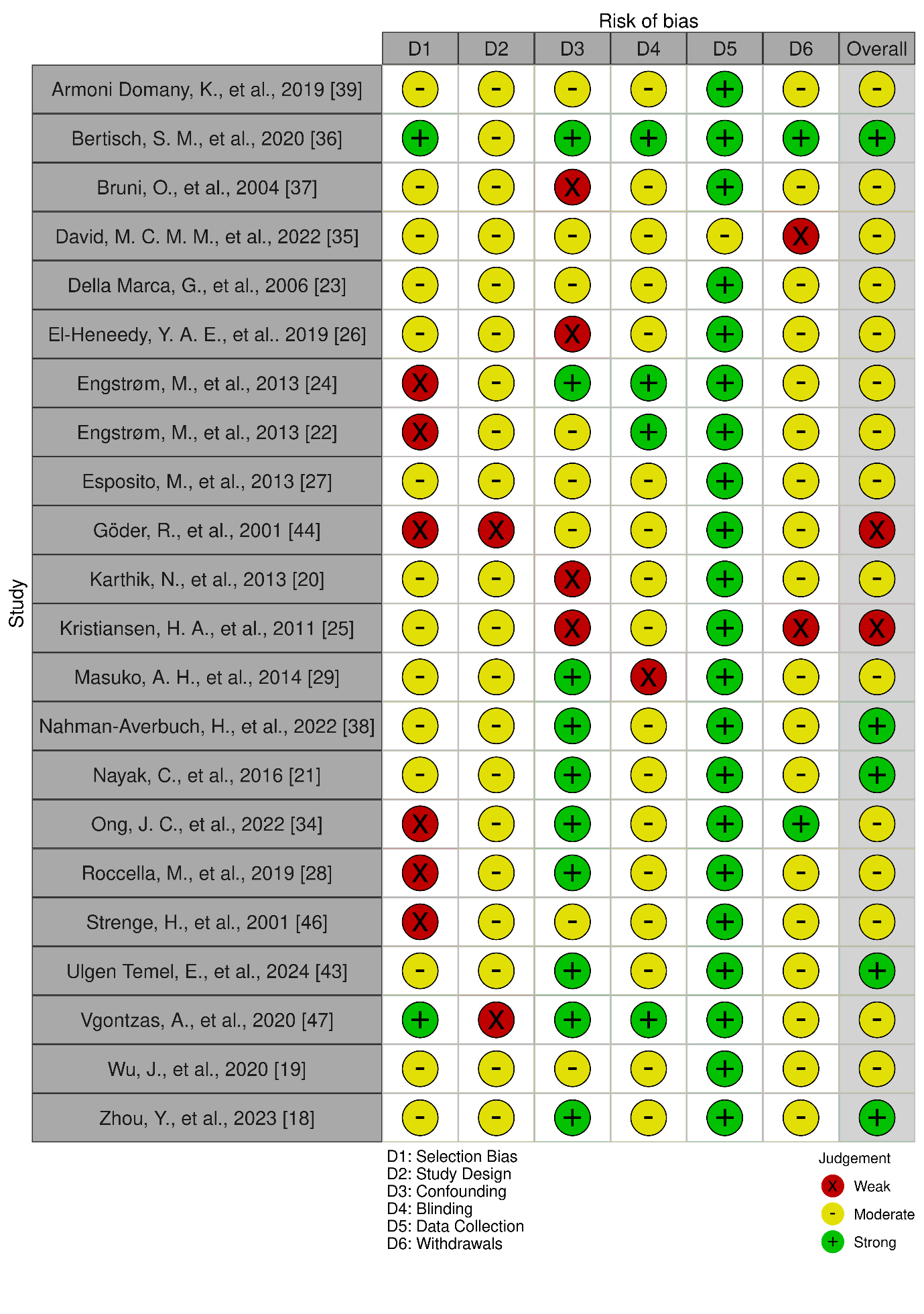


**Figure S1:** Risk of bias migraine studies. Summary of the risk of bias scores across studies of migraine patients, assessed using the Effective Public Health Practice Project quality assessment tool [15]. Figure created using robvis [73].


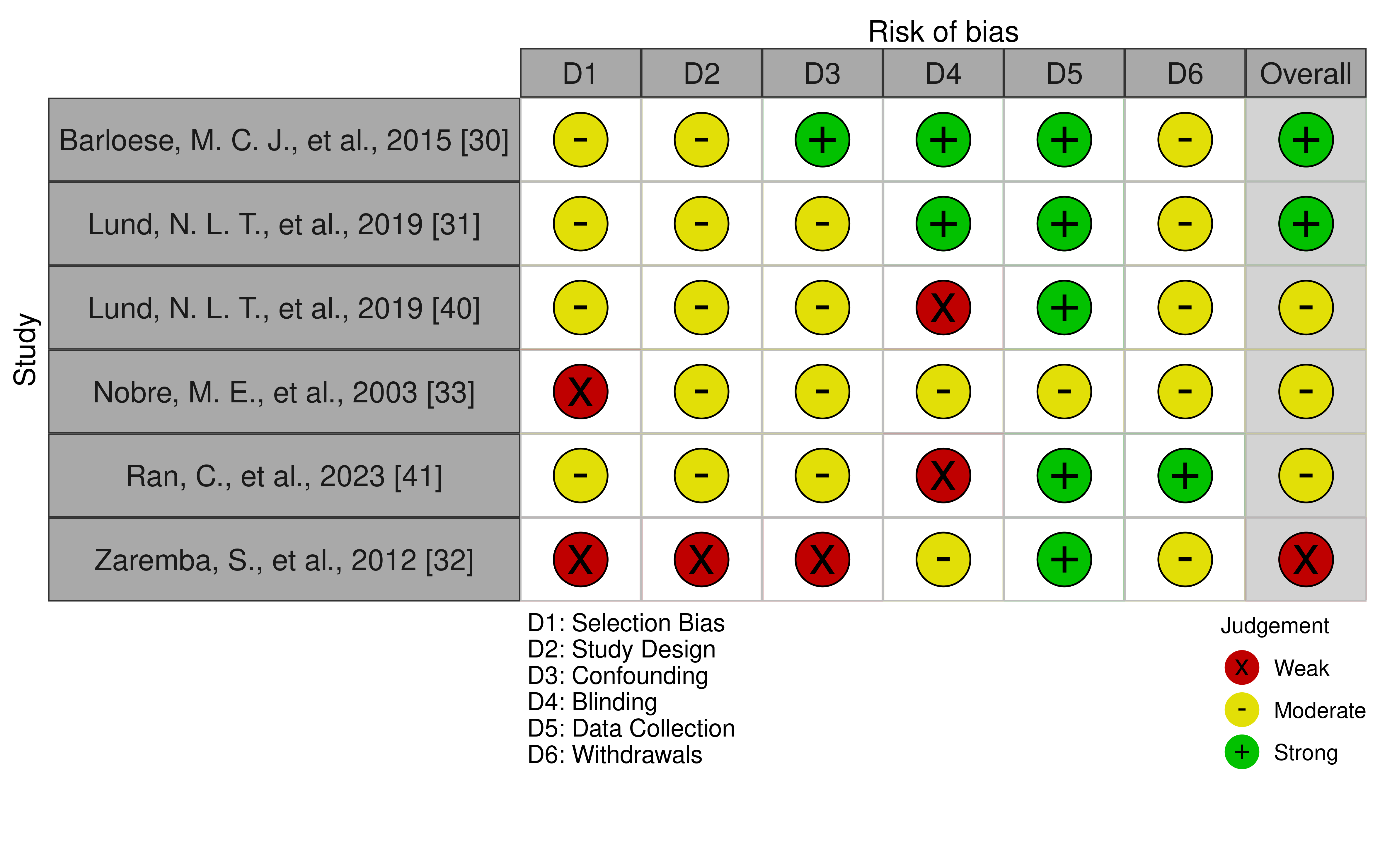


**Figure S2:** Risk of bias cluster headache. Summary of the risk of bias scores across studies of cluster headache patients, assessed using the Effective Public Health Practice Project quality assessment tool [15]. Figure created using robvis [73].

| **Bias** | **Domain** | Yu et al., 2023 [48] | Lillo-Vizin et al., 2024 [49] |
| --- | --- | --- | --- |
| Selection bias | Sequence generation | No | Unclear |
| Selection bias | Baseline characteristics | Yes | Yes |
| Selection bias | Allocation concealment | No | Unclear |
| Performance bias | Random housing | Unclear | Yes |
| Performance bias | Blinding | No | Unclear |
| Detection bias | Random outcome assessment | Unclear | Unclear |
| Detection bias | Blinding | Unclear | Yes |
| Attrition bias | Incomplete outcome data | Yes | No |
| Reporting bias | Selective outcome reporting | No | Unclear |
| Other | Other sources of bias | Crossover design in which all animals received same order of intervention. | NA |

**Table S6:**SYRCLE risk of bias assessment. Summary of the risk of bias assessment for the included animal model studies, assessed using SYRCLE [16].
